# Red Spot on the European Green Map: Will the Extra Catastrophic Phenomenon Take the Polish Poaching-Pressured Ospreys to the Brink of Extinction?

**DOI:** 10.3390/ani12010069

**Published:** 2021-12-29

**Authors:** Bartłomiej Woźniak, Michał Zygmunt, Łukasz Porębski, Patrycja Woźniak, Dariusz Anderwald

**Affiliations:** 1 Department of Forest Zoology and Wildlife Management, Institute of Forest Science, Warsaw University of Life Sciences—SGGW, Nowoursynowska 159, 02-776 Warsaw, Poland; 2 Eagle Conservation Committee, Jagiellończyka 45, 10-062 Olsztyn, Poland; m_zygmunt1@wp.pl (M.Z.); anderwaldd@gmail.com (D.A.); 3 General Directorate of the State Forests, Grójecka 127, 02-124 Warsaw, Poland; lukasz.porebski@lasy.gov.pl; 4 Independent Researcher, 02-796 Warsaw, Poland; patrycja.maniszewska@gmail.com

**Keywords:** Osprey, Poland, poaching, camera monitoring, unexpected extra mortality, predators, Northern Goshawk, Lifetime Reproductive Success, catastrophic extinction

## Abstract

**Simple Summary:**

Scientific research is an integral part of species protection. Thanks to the use of camera monitoring during a conservation project targeting a threatened Polish population of Osprey, we discovered that the influence of natural causes of brood losses is stronger than in previous years. This would be a kind of catastrophe for the species in the whole country. We conclude that only active protection and stopping the anthropogenic causes of Osprey mortality (e.g., poaching) could stop the decline in the population and give Poland a chance to not to be red spot on the European green map of Osprey.

**Abstract:**

Poland is the only European country where the Osprey population is declining due to the mortality of adult birds from poaching, which impacts not only single breeding attempts but also the Lifetime Reproductive Success (LRS) of specimens. However, what if there came an extra mortality factor in the moment of the lowest numbers of Osprey, already vulnerable in the country? In the years 2018–2020, we installed 22 trail cameras and five digital cameras (live online video feeds) on the nests. The total failure level observed in cameras (18.5%) was high. We observed, using these cameras, the extra mortality of chicks (10.7% of potentially fledged chicks) and even adult birds by unexpected predation by Northern Goshawk and White-tailed Eagle. This phenomenon is also common in the national population, as we found a total of ten cases of total losses by predators (eight or nine of them were birds of prey), including nests not covered by camera monitoring. The extra adult-predation by Goshawks means an extra drop in LRS. Those adult and chick predations are an example of exceptional catastrophic phenomena, which have been described as the direct cause of the extinction of animal populations throughout history. Only active conservation and stop poaching of the Polish population could stop the decline and save the Polish Ospreys.

## 1. Introduction

The Osprey (*Pandion haliaetus*) is one of the most widespread birds in the world [1]. After the poaching and Dichlorodiphenyltrichloroethane (DDT) catastrophe and consequent steep decline until 1970, the population has constantly increased since 1980, almost in the whole European breeding range. The populations in central European countries had the same trends and are high in Poland's neighboring countries today (700 pairs in Germany and 150–180 pairs in Belarus) [1,2]. In Poland, we observe the opposite situation and this country is the red spot on a green map of Ospreys in Europe. The Polish population is probably the lowest in at least 40 years, the number of breeding pairs are lower than 25, and the species has a vulnerable status in the country [3]. The main reason of this special situation is poaching of adult birds, especially the specimens, which use fish farming ponds as hunting grounds [4,5]. The small population is divided into two subpopulations, and we do not know if there is any real isolation between both, but it is very unlikely. The area of the western subpopulation is concentrated around the tri-border of Lubuskie, Wielkopolskie, and West-Pomeranian Voivodship. The eastern subpopulation inhabits the forested area of Mazury Lakeland [2,3]. Most Polish Ospreys breed in several areas of the Special Protection Areas (SPAs) Nature 2000 list, and only a few breed outside SPAs in Lubuskie Voivodship (W Poland) and in Romincka Forest (N-E Poland).

The survival of the threatened and very small population is at a greater risk as their eventual extinction may be directly attributed to a unique previously unobserved or previously not significant factor. This extra factor could be the direct cause of the extinction of the entire species, including local subpopulations [6,7]. What if such a factor occurred in the threatened Polish Osprey population? For example, predation is normal in all wild populations of birds. Since poaching caused the number of Ospreys to drop dramatically, every case of mortality of specimens, especially adults, could be a catastrophe for the national population. In Estonia, incidents of predation by Northern Goshawk (*Accipiter gentilis*) on Osprey chicks have been observed for a long time [8]. We did not observe this in Poland earlier, but it is possible that it has taken place here too. What if the predation of Goshawks and other predators is found to be unexpectedly high? That possible scenario could be a catastrophe for the Polish population of Osprey even if the main reason for decline is poaching [4,5].

In this paper, we will show the unexpected mortality of hatchlings, juveniles, and adults observed with the use of cameras and the potential impact of extra brood losses on the extremely threatened Polish population of the species.

## 2. Materials and Methods

In 2018–2020, Ltl Acorn Trail Cameras SGN-5220 (Ltl Acorn, Zhuhai, China) were installed near 22 Osprey nests in Poland. We also installed digital cameras near five nests to enable live online video feeds. Thus, we monitored all the nesting cycles of Ospreys in 27 nests from the prelaying period to the post-fledgling period. Video and trail cameras are widely used to monitor the nest breeding success and causes of offspring mortality [9,10,11,12]. In this study, we used the cameras to show the causes of nesting failures in Osprey nests in Poland and the potential impact of unusual extra mortality of chicks and adults on the vulnerable Polish population.

We also described the cases of “non-camera” mortality of chicks or fledglings found in the years 2018–2020 in the Polish population of Osprey and cases of mortality of adults and juveniles. We determined the causes of the mortality mainly from the traces, e.g., birds of prey nibbled on feathers, predatory mammals bit off feathers, or a bullet or bullet wound were found in poached birds. In one case, we observed a predator, which was destroying an Osprey brood. In one case, the mortality of juveniles occurred after leaving the camera monitored nest. The predator was established on the basis of traces and observations on camera (attempts to attack chicks during nesting period, visiting the nest after the juveniles left it). We did not use “non-camera” data in our analysis due to the insufficient accuracy of the collected data.

## 3. Results

**Figure 1:**
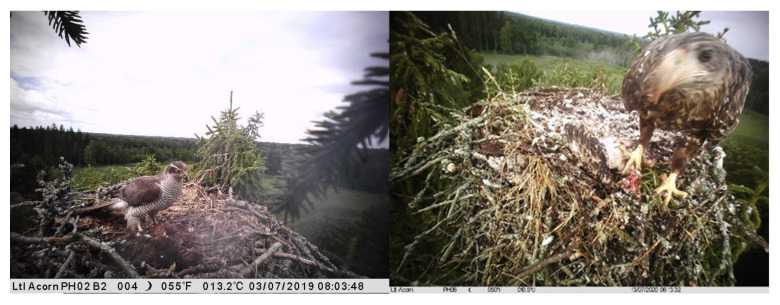
Northern Goshawk (**left**) and White-tailed Eagle (**right**) on the Osprey nests with hunted Osprey chicks.

In the years 2018–2020, five cases (18.5%) of total failure were found at nests monitored by cameras. Three of them were caused by predation on chicks, and the other two were the result of nonembryonic eggs or death of the embryos during incubation. In 2019, we recorded two cases of brood loss caused by Northern Goshawk predation. In 2020, we found the first documented case of predation by White-tailed Eagle (*Haliaeetus albicilla*) on the Osprey chicks in history (Figure 1, Table S1). In the two cases of egg failure, we observed secondary predation by Pine Marten (*Martes martes*) in one nest in 2019 and by Great Spotted Woodpecker (*Dendrocopos major*) and Eurasian Jay (*Garrulus glandarius*) in a second nest in 2020 (Table S1).

We found three cases (11.1%) of partial failure. In 2019, in one nest, one of the three eggs did not hatch and, in the second, one of the two did not hatch. In 2020, two of the three laid eggs were stolen by a predator, but we do not know the species of predator due to temporary damage to a trial camera (Table S1).

In summary, in the years 2018–2020, we found eight cases (29.6%) of failure (total plus partial) in camera-monitored nests, and predation was the cause of half of them. In the remaining half, the eggs did not hatch (Table S1). 

**Figure 2:**
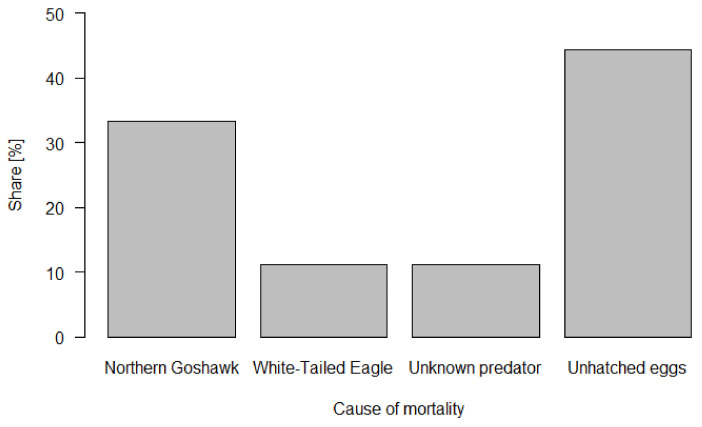
The share of causes of mortality of Osprey chicks in Poland determined on the basis of monitoring with the use of cameras (*n* = 18—number of dead chicks).

The possible totally fledgling number of all monitored nests could have been 75, but due to failures (total and partial), only 57 (76%) offspring fledged. Ten chicks and eggs (13.3% of potential production) were robbed by a predator. Six chicks were robbed by the Northern Goshawk (8% of potential production), two chicks (2.7%) by the White-tailed Eagle, and two eggs (2.7%) by an unidentified predator. Eight eggs (10.7%) did not hatch (Figure 2, Table S1).

We also observed the mortality of offspring and juveniles caused by predation in nests not monitored by cameras, all in eastern Poland. In 2019, we found three nest failures and mortality of juveniles from two nests in the post-fledging period. The Northern Goshawk was the raptor in three of them, including two territories in which five juveniles were hunted by members of this species (Table S1). In one nest, we did not know the number of offspring. In the second, the failure of a four chick brood was caused by an unknown bird of prey. In the last nest, one or two chicks were hunted but we do not know the species of the raptor. In 2020, we found two nest failures due to predation. One case was Pine Marten, in which we did not know the number of dead chicks. In one nest, we did not know the raptor, and one chick was hunted. 

In the entire country, using all the observations, we found that all Osprey nestlings/juveniles from 10 nests were hunted by predators. In five (50%) cases, the cause of the failure was Northern Goshawk; in one case (10%), it was White-tailed Eagle; in two cases, it was unknown birds of prey; in one (10%), Pine Marten; and in one, an unknown predator.

In one of the camera-monitored nests, we found predation by Northern Goshawk on adult birds. The male was hunted by the predator but also probably the female. We did not find the traces, but we did not see the female again. We also found three cases of poaching by humans of adults in the years 2018–2020. Two birds were dead, but one was injured, and after treatment, he returned to freedom.

## 4. Discussion

In this research, we observed the first case of predation by a White-tailed Eagle on Osprey chicks. Only in North America, the analogous species, the Bald Eagle (*Haliaeetus leucocephalus*) is described as a predator of Osprey’s nests [1,13], but the European *Haliaeetus* fish-eagle is not. The predation of Northern Goshawk is unusual too, even if Goshawks are nesting close to Ospreys. The year 2019 (plus 2020) could be unique because only in these years have Goshawks hunted Ospreys’ offspring and juveniles. We must remember that the cases of predation identified in this study may have been overlooked by researchers in the past, and only the use of trial cameras enabled the discovery of this phenomenon, e.g., in Estonia, cases of Northern Goshawk predation have been observed for years [8]. Even if this is the fact, it remains newly discovered information for Poland. On the other hand, brood losses due to Northern Goshawk predation were also detected in 2019–2020 by the traditional method of nest inspection without the use of cameras. There were also other cases of probable Goshawk (or White-tailed Eagle) predation, but the species has not been precisely established, as it is not always possible in the field. During many years of monitoring the species, such situations did not occur. Moreover, a significant decrease in Osprey breeding success has been observed in recent years [14,15]. That is why the phenomenon was certainly also known in previous years, but its scale was not so great. Similar events were confirmed in 2019–2021 in other European countries, where nests are monitored by cameras, e.g., Finland [16] and Latvia [17,18]. Ospreys actively defend their nests from predators, which is effective especially in populations that show high densities and even semicolonial breeding systems [1,19,20]. In situations where the density is higher, the Nearest Neighbor Distance (NND) is lower. Adult Ospreys that attack the predator in order to protect their chicks can also provide an umbrella for the chicks from neighboring nests. Additionally, by screaming, they can inform other Ospreys nesting nearby who are in the hunting grounds, and then such group attacks can be more effective. In Poland, the NND is higher due to the low number of breeding pairs [2,3,4,5], which gives the predators more free time to attack than in previous years when the population numbers were higher. Thus, in conclusion, the poaching, which is the main factor of the declining Osprey population [4,5], could be a reason why the cases of unusual extra predation have become more frequent. This cascade is very significant for Polish Ospreys.

The extra mortality of adults is even worse than nest failures. Most important for long-lived birds is not the one-year breeding success but the number of chicks produced during a lifetime, the Lifetime Reproductive Success (LRS) [21,22]. We confirmed the mortality of adults due to poaching. Until this research, we did not know the phenomenon of the extra mortality of adult Osprey due to Northern Goshawk predation. Even though the detectability is probably low and the predation has a small share in mortality, not only is the LRS of poached birds likely to suffer, but the LRS of hunted adults would also drop.

If we were to use only the internal criterion, the Polish osprey population should be classified as endangered but, due to high numbers of German and Belarusian populations and the possibility of supplying the Polish population with birds from neighboring countries, the category was upgraded to vulnerable [3]. If it were not for this extra supply, the rate of population decline in Poland could be even greater due to illegal hunting [4,5], and the species would be considered at least endangered. In this case, the extra mortality of adults and chicks due to unusual predation could affect the local Polish population and increase population decline, especially when more adults have lower LRS (due to poaching and extra predation), which must influence the very low population numbers. Throughout history, exceptional catastrophic phenomena were the direct cause of the species extinction even if the original cause bringing the species to the brink of extinction was different [6,7]. The extra mortality, especially of adults (high drop in LRS), but also of chicks in Poland could represent an instance of this type of catastrophe for the country’s population. It seems that only active protection focused on stopping poaching can lead to saving the national population and may make Poland turn green on the Osprey map of Europe.

## Data Availability

Camera films and live reports are available at the link: https://www.youtube.com/channel/UCzSO1yL2zJGHYwgzeBaVacg/videos (accessed on 22 December 2021); images and videos are partially published on the profile: https://www.facebook.com/RybolowyOnline (accessed on 22 December 2021).

## Supplementary Materials

The following is available online at https://www.mdpi.com/article/10.3390/ani12010069/s1. Table S1: Brood effects in single camera monitored nests of Osprey in Poland 2018–2020.
